# The Influence of Orthographic Units Across Korean Children of Different Ages in Hangul Reading

**DOI:** 10.3389/fpsyg.2022.797874

**Published:** 2022-04-01

**Authors:** Yeongsil Ju, Ami Sambai, Akira Uno

**Affiliations:** ^1^Department of Speech, Language, and Hearing Therapy, Faculty of Health Sciences, Mejiro University, Saitama, Japan; ^2^LD/Dyslexia Centre, Chiba, Japan; ^3^Faculty of Human Sciences, University of Tsukuba, Tsukuba, Japan

**Keywords:** reading, length, lexicality, frequency, non-lexical route, lexical route

## Abstract

Using the dual-route reading model as a framework, this study investigated the following research questions on Hangul reading: Which orthographic units (e.g., letters, syllable blocks, and words) influence the reading performance of Korean-speaking children? In addition, do the influential units change as the children grow up? To answer these questions, we tested the effects of age, frequency, lexicality, and two types of length—the numbers of letters (letter length) and syllable blocks (syllable block length)—and the interactions of these factors in the reading performance of Korean-speaking preschool and primary school children from first to third grade. Regarding reading latencies, there was a significant three-way interaction of age × lexicality × length regardless of the type of length. This interaction indicated that, for words only, the interaction between age and length was significant. Accordingly, the length effect decreased as children’s age increased. When reading latencies for words were analyzed with a mixed-effect model consisting of three factors—age, frequency, and length—neither a main effect of syllable block length nor an interaction of syllable block length with age was significant. In contrast, the interaction of age × letter length in word reading latencies remained significant. The length effect was smaller as children’s age increased. In addition, the frequency effect was significant and interacted significantly with age. The frequency effect increased as children’s age increased. In conclusion, significant frequency effects indicate that Korean-speaking children use the lexical process in addition to the non-lexical process when reading Hangul words. Importantly, as children grow up, a larger orthographic unit, that is, words, is more strongly related to reading performance, whereas the influence of the smaller orthographic unit, that is, letters, decreases.

## Introduction

Word attributes such as regularity, frequency, lexicality, and length influence the reading-aloud performance of adults in both alphabetic and non-alphabetic languages (e.g., Weekes, 1997; Bates et al., 2001; Juphard et al., 2004; Sambai et al., 2014; Rau et al., 2015). The effects of these attributes are important for elucidating the reading process. Several reading models (e.g., the triangle, dual-route cascaded, and connectionist dual models) have been suggested based on these attributes’ effects, especially in alphabetic languages. Moreover, the framework of these models has also been used to explain reading development. One such influential and widely used reading model is the dual-route cascaded model (Coltheart et al., 2001). In this study, we investigated the Hangul reading process of Korean-speaking children from the framework of the dual-route cascaded model (Coltheart et al., 2001).

### Dual-Route Reading Processes of Adults and Children

The dual-route cascaded model assumes two qualitatively different reading processes: a non-lexical route and a lexical route. In the non-lexical route, constituent graphemes of a word are sequentially converted to phonemes, whereas in the lexical route, a printed word is read aloud using lexical information such as spelling, sound, and meaning of words. Parallel processing is believed to occur *via* these routes (Coltheart et al., 2001).

The effects of lexicality and frequency are indicators of the use of the lexical route (Bates et al., 2001; Cuetos and Suárez-Coalla, 2009). Generally, the efficiency of the lexical route depends on word frequency; when using the lexical route, people read high-frequency words more accurately and rapidly than low-frequency words. In addition, words are read more correctly and rapidly than non-words, a phenomenon known as the lexicality effect. This phenomenon occurs because words are read correctly by both lexical and non-lexical routes, whereas non-words are processed correctly only by the non-lexical route.

Moreover, words and non-words with many letters are read less accurately and more slowly than those with fewer letters, which is called the length effect. The length effect is notably seen in reading non-words rather than words (Weekes, 1997). Given that non-words are read by the non-lexical route but not the lexical route, the length effect is an indicator of the use of the non-lexical route (Juphard et al., 2004; Rastle et al., 2009). Generally, the influence of the non-lexical route varies as a function of the efficiency of the lexical route (Weekes, 1997; Bates et al., 2001; Burani et al., 2002). Therefore, the magnitude of the length effect interacts with frequency; the length effect is larger for low-frequency words than for high-frequency words (Rastle et al., 2009). Examining the interaction between frequency and length in reading words aloud, in addition to the interaction between length and lexicality, will further elucidate the reading process, especially the efficiency of the lexical route for words.

Word attribute effects are also useful when exploring the reading process in children. According to research on reading development in transparent orthographies such as Italian and Spanish (Zoccolotti et al., 2005, 2009; Cuetos and Suárez-Coalla, 2009), length strongly influences the reading performance of children in the early stages of reading development. However, the length effect decreases as children advance in grade levels. This decrease is seen only in relation to words; for non-words, the length effect has been observed even in higher grade levels. As the length effect arises from the non-lexical route (Coltheart et al., 2001), children in the early stage read words predominantly *via* the non-lexical route and then gradually reduce their reliance on the non-lexical route as they accumulate reading experience (Zoccolotti et al., 2005; Sambai et al., 2012).

### The Writing System of Hangul

When reading Korean words, adults use both lexical and non-lexical routes (Yi and Lee, 1996). The Korean writing system, called Hangul, is alpha-syllabic. It consists of 14 consonant and 10 vowel symbols, with 16 compound letters derived from the basic letters (Taylor and Taylor, 1995; Cho et al., 2008). Hangul is fundamentally alphabetic. The smallest units in Korean phonology are phonemes. Each letter corresponds to a phoneme. In addition, the combination of multiple letters forms a block that corresponds to a syllable. Thus, letters are written in syllable blocks composed of a maximum of four graphemes (an example syllable composed of three graphemes is: ㄱ/k/+ㅗ/o/+ㅁ/m/→곰/kom/, bear). Hangul is a transparent orthography and is read by applying a one-to-one mapping between letters and sounds. In primary school education, children are taught how to sound out each letter (corresponding to phonemes) as well as how to pronounce each block (corresponding to syllables). Therefore, they may develop knowledge of orthography-to-phonology correspondences based on the letter-level and syllable block-level.

### Length Effects in Hangul Reading

In addition to the frequency effect, the length effect was observed in the Hangul reading of adults (Yi and Lee, 1996; Nam et al., 1997). Nam et al. (1997) investigated the effects of three types of length on adults’ reading performance. Since Korean spelling is decomposed into sub-lexical units such as letters, phonemes, and syllables, they defined word length based on the numbers of these units. For example, the word “엄마” /ʌm-ma/ (meaning mother in English) consists of two syllables (i.e., “엄”/ʌm/ and “마”/ma/”). In other words, the syllable length of this word is two. This word is composed of four phonemes: “ㅓ”/ʌ/, “ㅁ”/m/, “ㅁ”/m/, and “ㅏ”/a/. Therefore, the phoneme length is four. However, the word has five letters, “ㅇ, ㅓ, ㅁ, ㅁ, and ㅏ.” This is because, in the case of a syllable that starts with a vowel in Hangul, the dummy consonant (i.e., “ㅇ”) that is not sounded out is written before the vowel. Therefore, the letter length of “엄마” /ʌm-ma/ is five. In this way, the letter length and phoneme length may not necessarily match.

Nam et al. (1997) found that each type of length (i.e., letter length, phoneme length, and syllable length) affects the reading performance of adults. These results suggest that adults translate letters and syllable blocks into corresponding sounds. Therefore, it is assumed that the non-lexical reading process occurs for each letter and each syllable block during the reading of Hangul. In addition, Nam et al. (1997) reported that the effects of both letter length and syllable length interact with frequency.

Compared to adults’ reading, few studies investigate a length effect on Korean-speaking children’s Hangul reading. We found only one study by Hwang and Choi (2011), who report that fourth graders with normal reading development showed a significant syllable length effect on response times in the lexical decision task. This result suggests that the non-lexical reading process occurs in the Hangul word recognition of children.

### The Present Study

Study of Hwang and Choi (2011) had at least four limitations. First, they did not conduct a reading-aloud experiment, although they investigated the length effect on lexical decisions. Following the framework of the dual-route cascaded model (Coltheart et al., 2001), a lexical decision is made based on the level of activation in the orthographic lexicon. Therefore, the use of a lexical decision task is not sufficient to elucidate lexical and non-lexical processes, and a reading-aloud experiment that investigates the length effect is necessary.

Second, according to Nam et al. (1997), not only syllable length but also letter length affects adults’ reading performance. However, there has been no investigation on the effect of letter length on children’s reading performance. Although Hwang and Choi (2011) found a significant syllable length effect on the lexical decision performance of children, letter length tends to increase as syllable length increases. Therefore, it is unclear whether the non-lexical reading process occurs for letters or for syllable blocks when children read words aloud. It is necessary to test how the number of letters and syllable blocks influence children’s reading performance.

Third, the participants in Hwang and Choi (2011) were limited to children in the fourth grade. Previous studies (Zoccolotti et al., 2005; Sambai et al., 2012) found that younger children showed a larger length effect than older children. This decrease in the length effect occurs because the lexical reading process is more efficient and the reliance on the non-lexical route is weaker as a child grows up. Such developmental changes were observed between the first and second grades in Italian (Zoccolotti et al., 2005) and in Japanese kana (Sambai et al., 2012). Owing to the lack of previous studies on Korean, it is unclear whether such a change occurs in Hangul reading. As in previous studies (e.g., Sambai et al., 2012), it is necessary to compare the size of length effects on word reading performance in children with a wide range of ages, at least from preschoolers to third graders.

Fourth, Hwang and Choi (2011) investigated only the length effect. In adult reading, there is a frequency effect as well as an interaction between length and frequency (Nam et al., 1997), which suggests that the influence of the non-lexical route strengthens as the efficiency of the lexical route lowers. Investigating these reading phenomena will lead to a better understanding of the reading processes in Korean-speaking children.

Based on the limitations of Hwang and Choi (2011), this study addresses the following research questions:

Which orthographic units (e.g., letters, syllable blocks, and words) influence the reading performance of Korean-speaking children? In addition, will the influential units change as the children grow up?

To answer these questions, we investigated the effects of length, lexicality, and frequency on the reading performance of children from preschool to third grade. As mentioned before, unlike Japanese and Chinese characters, syllables in Hangul are made up of multiple phoneme/grapheme units (Pae, 2018). Since each syllable is written with multiple letters, which form a block visually, this study treated letters (representing phonemes) and blocks (representing syllables) as the main orthographic units. In this study, following definition of the types of length of Nam et al. (1997), the number of blocks was referred to as the syllable block length, whereas the number of letters was referred to as the letter length. Regarding length effects, we tested two types, namely letter and syllable block length, in the Hangul reading of Korean children.

When children use the lexical route, a lexicality effect or a frequency effect will be observed; when children use the non-lexical route, a length effect will be observed. In addition, when children use the non-lexical process at the letter-level, letter length will influence their reading performance; when children use the non-lexical process at the syllable block-level, syllable block length will influence their reading performance. We hypothesize that younger children rely more on the non-lexical route based on letter units, whereas older children use lexical information more efficiently. If our hypothesis is correct, then as a child’s age increases, the frequency effect (indicating the lexical route) will grow larger while the length effect (indicating the non-lexical route) will become smaller. In particular, the effect of letter length will be evident in younger children because their orthographic representations are poorer than those of older children.

## Materials and Methods

### Participants

A total of 83 native Korean children participated in this study. Data were excluded for children with test performance below 1.5 standard deviations of the mean score on Raven’s Colored Progressive Matrices, which is a general intelligence test. Furthermore, data were excluded for children whose test performance on the Receptive and Expressive Vocabulary Test fell below 1.5 standard deviations of the mean score, as administered in part by Park and Uno (2015). Finally, the data of 74 students were analyzed [kindergartners (*n* = 19), first graders (*n* = 30), second graders (*n* = 12), and third graders (*n* = 13); Table 1]. In a following reading latency experiment, we confirmed that all participants could read each constituent syllable of stimuli.

**Table 1:** Numbers and mean ages (months) of the participants.

	Male	Female	Total	Mean monthly age (SD)
Kindergartners	10	9	19	73.5 (4.4)
First graders	6	24	30	88.5 (5.5)
Second graders	3	9	12	103.2 (3.5)
Third graders	4	9	13	112.3 (3.3)
Total	23	51	74	

### Stimuli

We collected nouns from eight Korean language textbooks for first and second graders published by the Ministry of Education, removing homonyms. A total of 108 words were selected as stimuli, using a list created by sorting nouns in the decreasing order of frequency of their appearance in textbooks. Word stimuli comprised a frequency ranging from 1 to 374, the number of letters ranging from 2 to 13, and the number of syllable blocks ranging from 1 to 5. A total of 108 non-words were produced as stimuli by randomly replacing the constituent syllables of the 108 words. The number of letters in non-word stimuli ranged from 2 to 14, and the number of syllable blocks, from 1 to 5.

### Procedure

Stimuli were presented, and data were collected on the screen (15.6 inch) using DMDX (Forster and Forster, 2003) of a notebook computer (Intel® Core ™ i5-3210M CPU, 2.5 GHz). The children were asked to read aloud as quickly and accurately as possible. Each reading-aloud response was recorded directly onto a hard disk drive from a headset microphone. Each stimulus was presented on the screen in 40 pt. MS Gothic font. After the start button was pressed, a fixation marker (+) was presented in the middle of the screen for 500 ms, following which a stimulus was presented for 3,000 ms. A 2,000-ms interval was provided until the next trial. The participants sat approximately 50 cm away from the display. Before the experiment, six practice trials were conducted. Stimuli were randomly presented such that stimuli featuring the same condition would not be presented more than three times in a row. A short break was given after every 27 trials.

### Analysis

Speech waveforms were monitored using Check Vocal, and where the waveforms began was manually determined. Statistical software R (ver. 4.1.0) was used for statistical processing. We used a logistic mixed-effects model (Jaeger, 2008) to analyze reading accuracy and a mixed-effect model (Baayen, 2008) for reading latency data. Age (in months), length, lexicality, and frequency were treated as fixed-effect factors. We used age, length, and frequency as continuous variables. In addition, we used lexicality as a categorical variable. Length is defined as letter length and syllable block length.

## Results

The reading-aloud data contained two stimuli (i.e., 특이 and 석유) whose overall correct rates were 50% or lower. These data were excluded from the analysis.

### Reading Accuracy

The rate of correct responses (including self-corrections) was calculated.

#### Interactions Between Length and Lexicality

We ran a logistic mixed-effect model (Jaeger, 2008) using the lme4 package in R to test the main effects of age, lexicality, and two types of length (i.e., syllable block length and letter length) and the interactions between these variables. Correctness in each trial was the dependent variable. Fixed-effect factors were centered and scaled. Participants and stimuli were treated as random effects.

Different logistic mixed-effect models with main effects and interactions of the fixed-effect factors were refitted through the model criticism procedure (Baayen, 2008). Models were compared using Akaike Information Criterion (AIC). Each (*N*) model was derived from the previous (*N*−1) model after removing an interaction or a main effect (Table 2). The model associated to the numerically lowest AIC was considered the best model fitting data, that is, model 6. However, the model showed multicollinearity since the highest variation inflation factor value was 10.652. Therefore, we tested the effects of syllable block length and letter length separately in the following.

**Table 2:** Comparison of mixed-effects regression models on reading accuracy: age × lexicality × length (best fitting model in bold).

Model		AIC
Model1	Age*Lexicality*Syllable Block-Length+ Age*Lexicality*Letter-Length + (1 | SubjectID) + (1 | StimulusID)	8,740.2
Model2	Age*Lexicality+Age*Syllable Block-Length+ Lexicality*Syllable Block-Length+Age*Lexicality*Letter-Length + (1 | SubjectID) + (1 | StimulusID)	8,739.4
Model3	Age*Lexicality+Age*Syllable Block-Length+Lexicality* Syllable Block-Length+Age*Letter-Length +Lexicality*Letter-Length + (1 | SubjectID) + (1 | StimulusID)	8,740.1
Model4	Age*Syllable Block-Length+Lexicality*Syllable Block-Length+ Age*Lexicality*Letter-Length + (1 | SubjectID) + (1 | StimulusID)	8,739.4
Model5	Age*Lexicality+Lexicality*Syllable Block-Length+ Age*Lexicality*Letter-Length + (1 | SubjectID) + (1 | StimulusID)	8,737.8
**Model6**	Age*Lexicality+Syllable Block-Length+Age*Lexicality* Letter-Length + (1 | SubjectID) + (1 | StimulusID)	**8,736.6**
Model7	Age+Lexicality+Syllable Block-Length+Age*Lexicality* Letter-Length + (1 | SubjectID) + (1 | StimulusID)	8,736.6
Model8	Lexicality+Syllable Block-Length+Age*Lexicality*Letter-Length + (1 | SubjectID) + (1 | StimulusID)	8,736.6
Model9	Syllable Block-Length+Age*Lexicality*Letter-Length + (1 | SubjectID) + (1 | StimulusID)	8,736.6
Model10	Age*Lexicality*Letter-Length + (1 | SubjectID) + (1 | StimulusID)	8,745.3

**Figure 1:**
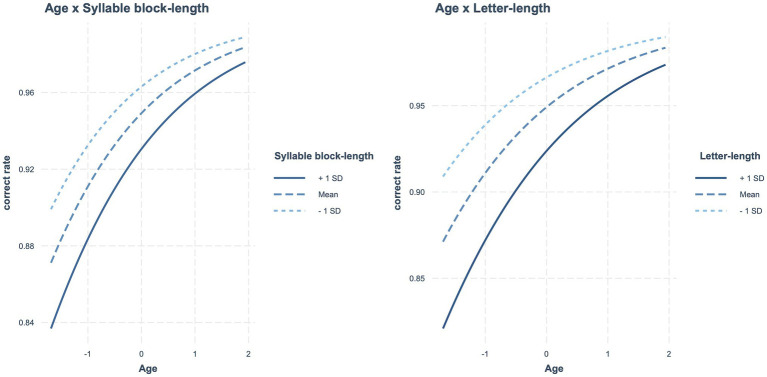
In reading accuracy, significant interactions between age and two types of length (i.e., syllable block length and letter length).

Table 3 presents the estimated fixed effects of syllable block length along with other factors. The main effects of age, lexicality, and syllable block length were significant. The syllable block length effect also interacted significantly with age. Accordingly, as age increased, the syllable block length effect decreased. The interaction between age and syllable block length is shown in Figure 1. No other significant interactions were observed.

**Table 3:** Estimates of fixed effects in reading accuracy: age × lexicality × length.

Fixed effects	Syllable block length				Letter length			
	*β*	SE	*z*	*p*	*β*	SE	*z*	*p*
(Intercept)	2.445	0.149	16.366	0.000	2.449	0.147	16.658	0.000
Age	0.599	0.107	5.591	0.000	0.604	0.107	5.627	0.000
Lexicality	0.978	0.163	6.006	0.000	0.969	0.158	6.127	0.000
Length	−0.401	0.108	−3.711	0.000	−0.486	0.105	−4.636	0.000
Age × Lexicality	0.005	0.064	0.076	0.939	−0.007	0.065	−0.102	0.918
Age × Length	−0.071	0.033	−2.134	0.033	−0.075	0.034	−2.244	0.025
Lexicality × Length	0.140	0.160	0.879	0.379	0.114	0.154	0.737	0.461
Age × Lexicality × Length	0.076	0.060	1.269	0.204	0.101	0.059	1.721	0.085

*The result of syllable block length is when the length is counted in syllable blocks and the result of letter length is when the length is counted in letters*.

Similar results were obtained when letter length was contained in a model instead of syllable block length (Table 3). The main effects of age, lexicality, and letter length were significant, as was the interaction between age and letter length. Accordingly, as age increased, the letter length effect decreased (Figure 1). No other significant interactions were observed.

#### Interactions Between Length and Frequency

To test the main effects of age, two types of length, and frequency and interactions between these variables in the accuracy of reading words, we ran a logistic mixed-effect model (Jaeger, 2008) using the lme4 package in R with correctness in each trial as the dependent variable. Fixed-effect factors were centered and scaled. Participants and stimuli were treated as random effects.

Different logistic mixed-effect models with main effects and interactions of the fixed-effect factors were refitted through the model criticism procedure (Baayen, 2008). Models were compared using AIC. Each (*N*) model was derived from the previous (*N*−1) model after removing an interaction or a main effect (Table 4). The model associated to the numerically lowest AIC was considered the best model fitting data, that is, model 1. However, the model showed multicollinearity since the highest variation inflation factor value was 14.107. Therefore, we tested the effects of syllable block length and letter length separately.

**Table 4:** Comparison of mixed-effects regression models on reading accuracy: age × frequency × length on words (best-fitting model in bold).

Model		AIC
**Model1**	Age*Frequency*Syllable Block-Length+Age*Frequency*Letter-Length + (1 | SubjectID) + (1 | StimulusID)	**3,398.1**
Model2	Age*Frequency + Age*Syllable Block-Length+Frequency*Syllable Block-Length+Age*Lexicality*Letter-Length + (1 | SubjectID) + (1 | StimulusID)	3,400.4
Model3	Age*Frequency*Syllable Block-Length+Age*Frequency+Age*Letter-Length+Frequency*Letter-Length + (1 | SubjectID) + (1 | StimulusID)	3,399.1
Model4	Age*Frequency*Syllable Block-Length+Age*Letter-Length+ Frequency*Letter-Length + (1 | SubjectID) + (1 | StimulusID)	3,399.1
Model5	Age*Frequency*Syllable Block-Length+Age*Frequency+ Frequency*Letter-Length + (1 | SubjectID) + (1 | StimulusID)	3,398.2
Model6	Age*Frequency*Syllable Block-Length+Age*Frequency+ Letter-Length + (1 | SubjectID) + (1 | StimulusID)	3,398.8
Model7	Age*Frequency*Syllable Block-Length+Age+Frequency+ Letter-Length + (1 | SubjectID) + (1 | StimulusID)	3,398.8
Model8	Age*Frequency*Syllable Block-Length+Age*Frequency + (1 | SubjectID) + (1 | StimulusID)	3,410.5

**Figure 2:**
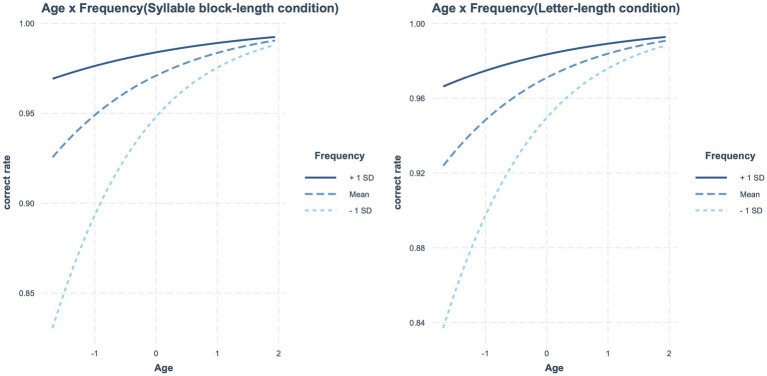
In reading accuracy, significant interaction between age and frequency.

Table 5 presents the estimated fixed effects of syllable block length along with other factors. The main effects of age and frequency were significant, but the syllable block length effect was not. The frequency effect interacted significantly with age. Accordingly, as age increased, the frequency effect decreased. The interaction between age and frequency is shown in Figure 2. No other significant interactions were observed.

**Table 5:** Estimates of fixed effects in reading accuracy: age × frequency × length on words.

Fixed effects	Syllable block length				Letter length			
	*β*	SE	*z*	*p*	*β*	SE	*z*	*p*
(Intercept)	3.502	0.168	20.884	0.000	3.504	0.164	21.305	0.000
Age	0.590	0.118	5.019	0.000	0.604	0.118	5.129	0.000
Frequency	0.602	0.134	4.489	0.000	0.572	0.128	4.456	0.000
Length	−0.079	0.129	−0.609	0.542	−0.196	0.122	−1.598	0.110
Age × Frequency	−0.190	0.061	−3.100	0.002	−0.167	0.060	−2.790	0.005
Age × Length	−0.082	0.062	−1.320	0.187	−0.034	0.058	−0.584	0.559
Frequency × Length	0.196	0.151	1.301	0.193	0.243	0.140	1.732	0.083
Age × Frequency × Length	−0.099	0.072	−1.378	0.168	−0.043	0.065	−0.658	0.511

*The result of syllable block length is when the length is counted in syllable blocks and the result of letter length is when the length is counted in letters*.

When letter length was contained in a model instead of syllable block length, the same results were obtained again (Table 5). In other words, the main effects of age and frequency were significant, but the effect of letter length was not. The interaction between age and frequency was significant, indicating that the frequency effect decreased as age increased (see Figure 2). No other significant interactions were observed.

### Reading Latency

The reading latencies available for statistical analyses were determined using the following procedure. In line with previous studies (Zoccolotti et al., 2005; Sambai et al., 2012), reading latencies (RTs) of incorrect or non-responses (10.9%) and of self-corrected responses (3.0%) were excluded, resulting in the exclusion of a total of 13.9% of the reading latencies.

#### Interactions Between Length and Lexicality

To test the main effects of age, lexicality, and two types of length and the interactions between these variables, we ran a mixed-effect model (Baayen, 2008) using the lme4 package in R with RTs in each trial as the dependent variable. Fixed-effect factors were centered and scaled. Participants and stimuli were treated as random effects. The trial number was included as a covariate.

Different mixed-effect models with main effects and the interactions of the fixed-effect factors were refitted through the model criticism procedure (Baayen, 2008). Models were compared using AIC. Each (*N*) model was derived from the previous (*N*−1) model after removing an interaction or a main effect (see Table 6). The model associated with the numerically lowest AIC was considered the best model fitting data, that is, model 6. However, the model showed multicollinearity since the highest variation inflation factor value was 10.342. Therefore, we tested the effects of syllable block length and letter length separately.

**Table 6:** Comparison of mixed-effects regression models on reading latency: age × lexicality × length (best-fitting model in bold).

Model		AIC
Model1	Age*Lexicality*Syllable Block-Length + Age*Lexicality*Letter-Length + (1|SubjectID) + (1|StimulusID)	−183,563
Model2	Age*Lexicality + Age*Syllable Block-Length + Lexicality*Syllable Block-Length + Age*Lexicality*Letter-Length + (1|SubjectID) + (1|StimulusID)	−183,565
Model3	Age*Lexicality + Age*Syllable Block-Length + Lexicality*Syllable Block-Length + Age*Lexicality + Age*Letter-Length + Lexicality*Letter-Length + (1|SubjectID) + (1|StimulusID)	−183,559
Model4	Age*Syllable Block-Length + Lexicality*Syllable Block-Length + Age*Lexicality*Letter-Length + (1|SubjectID) + (1|StimulusID)	−183,565
Model5	Age*Lexicality + Age*Syllable Block-Length + Age*Lexicality*Letter-Length + (1|SubjectID) + (1|StimulusID)	−183,566
**Model6**	Age*Lexicality + Syllable Block-Length + Age*Lexicality*Letter-Length + (1|SubjectID) + (1|StimulusID)	**−183,567**
Model7	Age + Lexicality + Syllable Block-Length + Age*Lexicality*Letter-Length + (1|SubjectID) + (1|StimulusID)	−183,567
Model8	Age*Lexicality + Age*Lexicality*Letter-Length + (1|SubjectID) + (1|StimulusID)	−183,566

**Figure 3:**
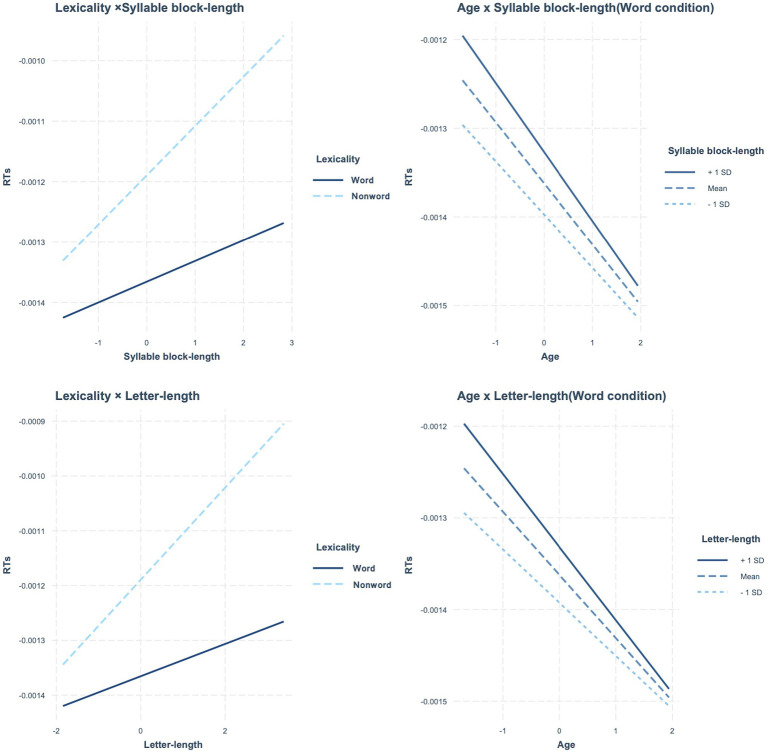
In reading latency, significant interactions between lexicality and two types of length, and between age and length. **Left panels**: interaction between lexicality and two types of length. **Right panels**: interaction between age and length in word condition, related to the three-way interaction.

Table 7 presents the estimated fixed effects of syllable block length along with other factors. The main effects of age, lexicality, and syllable block length were significant. There was a significant interaction between lexicality and syllable block length. In addition, the three-way interaction of age × lexicality × length was significant. No other significant interactions were observed. Since the three-way interaction was significant, we tested the main effect of age and syllable block length and the interaction of these variables per lexicality. In word reading, the main effects of age and syllable block length were significant (age: *β* = −0.000, SE = 0.000, *t* = −3.102, *p* = 0.003; syllable block length: *β* = 0.000, SE = 0.000, *t* = 2.935, *p* = 0.004). The interaction of these variables was also significant; as age increased, the effect of syllable block length decreased (*β* = −0.000, SE = 0.000, *t* = −2.617, *p* = 0.009). In non-word reading, the main effects of age and syllable block length were significant, but the interaction of these variables was not (age: *β* = −0.000, SE = 0.000, *t* = −2.652, *p* = 0.010; syllable block length: *β* = 0.000, SE = 0.000, *t* = 7.561, *p* < 0.001; interaction: *β* = 0.000, SE = 0.000, *t* = 0.733, *p* = 0.463). Figure 3 presents the significant interactions.

**Table 7:** Estimates of fixed effects in reading latency: age × lexicality × length.

Fixed effects	Syllable block length				Letter length			
	*β*	SE	*t*	*p*	*β*	SE	*t*	*p*
(Intercept)	−0.001	0.000	−48.208	0.000	−0.001	0.000	−48.213	0.000
Age	−0.000	0.000	−2.775	0.007	−0.000	0.000	−2.774	0.007
Lexicality	−0.000	0.000	−10.915	0.000	−0.000	0.000	−10.926	0.000
Length	0.000	0.000	7.120	0.000	0.000	0.000	7.340	0.000
Age × Lexicality	−0.000	0.000	−1.266	0.206	−0.000	0.000	−1.298	0.194
Age × Length	0.000	0.000	0.747	0.455	0.000	0.000	0.843	0.399
Lexicality × Length	−0.000	0.000	−2.889	0.004	−0.000	0.000	−3.344	0.001
Age × Lexicality × Length	−0.000	0.000	−2.311	0.021	−0.000	0.000	−2.761	0.006

*The result of syllable block length is when the length is counted in syllable blocks and the result of letter length is when the length is counted in letters*.

When the letter length was included in a mixed-effect model instead of the syllable block length, we obtained results similar to when testing for the effect of the syllable block length discussed above (Table 7). The main effects of age, lexicality, and letter length were significant. There were significant lexicality × letter length and age × lexicality × letter length interactions. Since the three-way interaction was significant, we tested the main effects of age and letter length and the interaction of these variables per lexicality. In word reading, the main effects of age and letter length were significant, as was the interaction (age: *β* = −0.000, SE = 0.000, *t* = −3.108, *p* = 0.003; letter length: *β* = 0.000, SE = 0.000, *t* = 2.514, *p* = 0.013; interaction: *β* = −0.000, SE = 0.000, *t* = −3.184, *p* = 0.001; see Figure 3). In non-word reading, the main effects of age and letter length were significant, but the interaction was not (age: *β* = −0.000, SE = 0.000, *t* = −2.650, *p* = 0.010; letter length: *β* = 0.000, SE = 0.000, *t* = 7.907, *p* < 0.001; interaction: *β* = 0.000, SE = 0.000, *t* = 0.878, *p* = 0.380).

#### Interactions Between Length and Frequency

To test the main effects of age, two types of length, and the frequency and interactions between these variables in the accuracy of reading words, we ran a mixed-effect model (Baayen, 2008) using the lme4 package in R with RTs in each trial as the dependent variable. Fixed-effect factors were centered and scaled. Participants and stimuli were treated as random effects. The trial number was included as a covariate.

Different mixed-effect models with main effects and interactions of the fixed-effect factors were refitted through the model criticism procedure (Baayen, 2008). Models were compared using AIC. Each (*N*) model was derived from the previous (*N*−1) model after removing an interaction or a main effect (see Table 8). The model associated to the numerically lowest AIC was considered the best model fitting data, that is, model 3. However, the model showed multicollinearity since the highest variation inflation factor value was 9.644. Therefore, we tested the effects of syllable block length and letter length separately.

**Table 8:** Comparison of mixed-effects regression models on reading latency: age × frequency × length on words (best-fitting model in bold).

Model		AIC
Model1	Age*Frequency*Syllable Block-Length + Age*Frequency*Letter-Length + (1|SubjectID) + (1|StimulusID)	−95,788
Model2	Age*Frequency + Age*Syllable Block-Length + Frequency*Syllable Block-Length + Age*Frequency*Letter-Length + (1|SubjectID) + (1|StimulusID)	−95,788
**Model3**	Age*Frequency + Age*Syllable Block-Length + Frequency*Syllable Block-Length + Age*Letter-Length + Frequency*Letter-Length + (1|SubjectID) + (1|StimulusID)	**−95,790**
Model4	Frequency*Syllable Block-Length + Age*Syllable Block-Length + Age *Letter-Length + Frequency*Letter-Length + (1|SubjectID) + (1|StimulusID)	−95,787
Model5	Age*Frequency + Age*Syllable Block-Length + Age*Letter-Length + Frequency*Letter-Length + (1|SubjectID) + (1|StimulusID)	−95,788
Model6	Age*Frequency + Frequency*Syllable Block-Length + Age*Letter-Length + Frequency*Letter-Length + (1|SubjectID) + (1|StimulusID)	−95,790
Model7	Age*Frequency + Frequency*Syllable Block-Length + Age*Letter-Length + (1|SubjectID) + (1|StimulusID)	−95,790
Model8	Age*Frequency + Frequency*Syllable Block-Length + Letter-Length + (1|SubjectID) + (1|StimulusID)	−95,786
Model9	Age + Frequency*Syllable Block-Length + Age*Letter-Length + (1|SubjectID) + (1|StimulusID)	−92,774
Model10	Age*Frequency + Syllable Block-Length + Age*Letter-Length + (1|SubjectID) + (1|StimulusID)	−92,816

**Figure 4:**
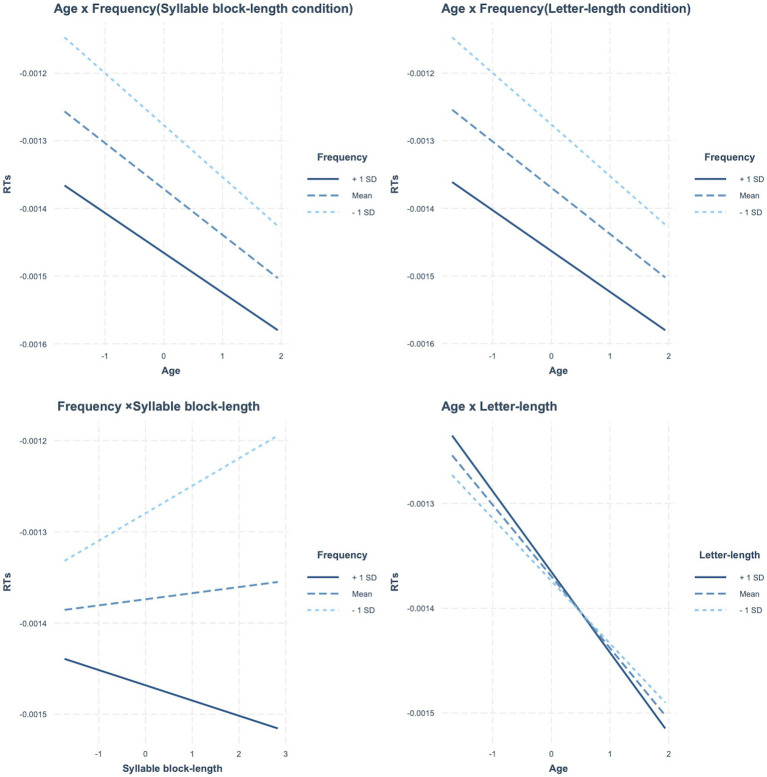
In reading latencies for words, significant interactions. **Top panels**: interaction between age and frequency in two type of length conditions. **Bottom panels**: interaction between frequency and syllable block length (left), age and letter length (right).

Table 9 presents the estimated fixed effects of syllable block length along with other factors. The main effects of age and frequency were significant, but the syllable block length effect was not. The frequency effect interacted significantly with age. Accordingly, as age increased, the frequency effect became stronger. The interaction between syllable block length and frequency was also significant. No other significant interactions were observed. Figure 4 presents these significant two-way interactions.

**Table 9:** Estimates of fixed effects in reading latency: age × frequency × length on words.

Fixed effects	Syllable block length				Letter length			
	*β*	SE	*t*	*p*	*β*	SE	*t*	*p*
(Intercept)	−0.001	0.000	−57.330	0.000	−0.001	0.000	−57.164	0.000
Age	−0.000	0.000	−3.075	0.003	−0.000	0.000	−3.100	0.003
Frequency	−0.000	0.000	−9.994	0.000	−0.000	0.000	−9.858	0.000
Length	0.000	0.000	0.917	0.361	0.000	0.000	0.589	0.557
Age × Frequency	0.000	0.000	2.418	0.016	0.000	0.000	2.173	0.030
Age × Length	−0.000	0.000	−1.748	0.081	−0.000	0.000	−2.466	0.014
Frequency × Length	−0.000	0.000	−2.226	0.028	−0.000	0.000	−1.711	0.090
Age × Frequency × Length	0.000	0.000	1.039	0.299	0.000	0.000	0.610	0.542

*The result of syllable block length is when the length is counted in syllable blocks and the result of letter length is when the length is counted in letters*.

Table 9 also presents the estimated fixed effects of letter length along with other factors. The main effects of age and frequency were significant, but the letter length effect was not. The effects of frequency and letter length interacted significantly with age. Accordingly, as age increased, the frequency effect became stronger. In contrast, the letter length effect became smaller with an increase in age. Figure 4 presented these significant two-way interactions. No other significant interactions were observed.

## Discussion

We hypothesized that reading performance would be affected more strongly by a process involving larger orthographic units as children grow up. If this hypothesis is true, the effect of word frequency (i.e., a lexical variable) on reading performance would be larger, whereas the effect of length (i.e., a sub-lexical variable) would be smaller as a child’s age increases.

The results of the children’s RTs support our hypothesis. There was a significant three-way interaction of age × lexicality × length regardless of length type (i.e., syllable block length or letter length). The interaction indicated that the length effect on RTs became smaller as children’s age increased in reading words rather than non-words. This result was consistent with previous studies in alphabetic languages, which showed a decrease in the length effect on word reading performance (Zoccolotti et al., 2005, 2009; Cuetos and Suárez-Coalla, 2009). Importantly, when a mixed-effect model included frequency, both the significant main effect of syllable block length and a significant interaction of syllable block length × age disappeared in word reading. However, the frequency effect was significant. Given that frequency is a variable showing the involvement of a lexical reading process, while length is a variable showing the involvement of a non-lexical reading process (Weekes, 1997; Bates et al., 2001; Burani et al., 2002), it is supposed that our participants read words predominantly using a lexical rather than a non-lexical process in which syllable blocks are converted to corresponding sounds. In contrast to there being no syllable block length effect, even when a mixed-effect model included frequency, the letter length effect interacted negatively with age. This indicates that a non-lexical process based on smaller orthographic units (i.e., letters) still influenced the reading performance of younger children in particular. In addition, since a positive interaction of frequency × age was significant in a mixed-effect model with the syllable block or letter length factor, it is likely that a process involving a bigger orthographic unit (i.e., words) is more strongly related to the word reading performance of older children.

Regarding reading accuracy, the effects of letter length and syllable block length were significant when the reading data of words and non-words were analyzed together using a mixed-effect model with the main effects of age, lexicality, and length and the interactions of these factors. In addition, both length effects were modulated by age; younger children tended to make mistakes more frequently as stimuli had more letters or syllable blocks. These results suggest that the application of letter(s)-to-sound(s) correspondence rules in younger children is more error-prone. However, neither the letter length effect nor the syllable block length effect was observed when only reading data of words were analyzed using a mixed-effect model with frequency as a fixed-effect factor.

In contrast, frequency significantly influences reading accuracy. Importantly, younger children showed a greater frequency effect than older children. This interaction of frequency and age in reading accuracy was in the opposite direction of the reading latency data. This result did not match our expected results regarding the influence of frequency on the reading performance of older children. However, we believe that these results do not contradict our hypothesis. Given that younger children tended to make mistakes more frequently than older children, they might have needed to compensate for their poor mappings between letter(s) and sound(s) using lexical knowledge, resulting in the larger frequency effect.

## Limitations

This study has some limitations. First, the sample size was small for each grade. Therefore, we could not identify the specific period during which the predominant reading process changed. Further studies should include more participants in each grade. Second, this was a cross-sectional study. A longitudinal study is necessary to clarify the developmental changes in the reading process in greater detail. Therefore, future research should include a longitudinal study with more participants.

## Conclusion

In this study, the dual-route cascaded model was applied to the reading of Hangul, and the reading process of Korean children was investigated. The study revealed the following: First, Korean-speaking children used not only non-lexical but also lexical reading processes when reading words. Second, as children’s ages increased, the influence of larger orthographic units became stronger. This study suggests that in the non-lexical route, Korean-speaking children use orthography-to-phonology mappings at two orthographic levels, that is, letters and syllable blocks. In other words, the non-lexical processing may comprise at least two decoding systems: a letter-based one and a syllable block-based one.

In terms of practical relevance, these results, based on the Korean writing system, may be useful for developing practical research for children who have developmental dyslexia. In addition, the results suggest that the influence of different orthographic units changes as a child grows up in transparent languages. It could have implications for consideration in other languages as well.

## Data Availability Statement

The raw data supporting the conclusions of this article will be made available by the authors, without undue reservation.

## Ethics Statement

The studies involving human participants were reviewed and approved by Faculty of Human Sciences, University of Tsukuba. Written informed consent to participate in this study was provided by the participants’ legal guardian/next of kin.

## Author Contributions

YJ was mainly responsible for data collection and analysis and completed the draft. AS was involved in the analysis and writing of parts of the manuscript. AU revised the manuscript. All authors contributed to the article and approved the submitted version.

## Conflict of Interest

The authors declare that the research was conducted in the absence of any commercial or financial relationships that could be construed as a potential conflict of interest.

## Publisher’s Note

All claims expressed in this article are solely those of the authors and do not necessarily represent those of their affiliated organizations, or those of the publisher, the editors and the reviewers. Any product that may be evaluated in this article, or claim that may be made by its manufacturer, is not guaranteed or endorsed by the publisher.

## Appendix

**Words**

책, 꽃, 곰, 발, 물, 뒤, 댁, 낫, 널, 득, 뼘, 꾀, 친구, 그림, 사람, 공부, 시간, 가족, 얼굴, 약속, 지금, 소리, 놀이, 마음, 노래, 이름, 누나, 엄마, 국어, 나무, 돼지, 우리, 아기, 고뿔, 꽁지, 소복, 기만, 올림, 지천, 밀고, 팔자, 가락, 다양, 석유, 양지, 고을, 대꾸, 미라, 자음, 특이, 누리, 무쇠, 구유, 누이, 선생님, 장난감, 인형극, 파란색, 그림책, 냉장고, 동물원, 운동장, 할머니, 목소리, 달리기, 물고기, 부모님, 자전거, 시험지, 자동차, 호랑이, 아줌마, 강아지, 무지개, 코끼리, 분실물, 순식간, 자명종, 다홍색, 말다툼, 시설물, 별안간, 냉방기, 수증기, 돌덩이, 광주리, 약봉지, 물보라, 구절초, 전나무, 털배자, 꾀꼬리, 시치미, 조바위, 영구치, 봉오리, 숨바꼭질, 텔레비전, 비눗방울, 그림일기, 우리나라, 산산조각, 발뒤꿈치, 치맛자락, 버들가지, 일기예보, 엄지손가락, 색동저고리

**Non-words**

넣, 겪, 른, 칭, 림, 려, 깥, 눗, 련, 늬, 쬐, 쩌, 엄구, 이림, 지람, 나부, 사간, 누족, 국굴, 우속, 마금, 가리, 얼이, 아음, 약래, 공름, 돼나, 그마, 노어, 소무, 놀지, 시리, 친기, 다뿔, 석지, 누복, 무만, 자림, 고천, 양고, 올자, 밀락, 구양, 팔유, 특지, 기을, 누꾸, 고라, 꽁음, 미이, 차리, 소쇠, 대유, 지이, 운생극, 선난색, 동형지, 호란거, 자림차, 기장냉, 자물리, 시동마, 목머이, 아소장, 달리책, 물고님, 코모감, 부전고, 할험님, 강동개, 무랑지, 장줌원, 파아기, 그지니, 인끼리, 광실라, 자식리, 꾀명색, 털홍위, 이다돌, 물설자, 수안툼, 시방간, 다증지, 시덩치, 순주기, 구봉미, 리보냉, 조절종, 기나봉, 별배물, 말꼬초, 분치리, 영바물, 약구무, 전오간, 일바예질, 산레자전, 버눗가울, 숨림방기, 그리꿈라, 텔산일각, 비뒤나치, 조맛치락, 발들비지, 우기꼭보, 리지색가저, 락동엄고손
